# The Clinical Implications of Serum Carbohydrate Antigen 19-9 Levels in Patients with Nontuberculous Mycobacteria Pulmonary Disease

**DOI:** 10.3390/jcm12247751

**Published:** 2023-12-18

**Authors:** Daegeun Lee, Byung Woo Jhun

**Affiliations:** 1 Division of Pulmonary, Allergy and Critical Care Medicine, Chung-Ang University Gwangmyeong Hospital, Gwangmyeong 14353, Republic of Korea; maildori@naver.com; 2 Division of Pulmonary and Critical Care Medicine, Department of Medicine, Samsung Medical Center, Sungkyunkwan University School of Medicine, 81 Irwon-ro, Gangnam-gu, Seoul 06351, Republic of Korea

**Keywords:** nontuberculous mycobacteria, carbohydrate antigen, outcome, treatment

## Abstract

Serum carbohydrate antigen 19-9 (CA19-9) levels can increase in nontuberculous mycobacteria pulmonary disease (NTM-PD), and the levels correlate with disease activity. We compared the clinical characteristics of NTM-PD patients with and without elevated CA19-9 levels and evaluated its association with antibiotic response in a retrospective study of NTM-PD patients diagnosed between January 1994 and December 2020. We analyzed 1112 patients who had serum CA19-9 measured: 322 with elevated CA19-9 and 790 with normal CA19-9. The erythrocyte sedimentation rate and C-reactive protein levels were significantly higher in the elevated CA19-9 group (*p* < 0.001 and *p* = 0.029, respectively). The 1-year culture conversion rate after antibiotics did not differ between the elevated (n = 206) and normal (n = 377) CA19-9 groups (80% vs. 72%, *p* = 0.055). Analysis of a subset of 434 patients revealed that current smoking, bronchiectasis, acid-fast bacilli smear positivity, and the *M. abscessus* strain significantly reduced microbiological cure rates. Serum CA 19-9 levels did not have a significant association with microbiological cure in a multivariate analysis. These findings suggest that the role of serum CA19-9 in predicting antibiotic treatment outcomes is limited, and that elevated CA19-9 does not necessarily indicate a poor outcome.

## 1. Introduction

Nontuberculous mycobacteria (NTM) refer to mycobacteria excluding *Mycobacterium tuberculosis* and *Mycobacterium leprae*, and they are ubiquitous organisms found in the environment [1,2]. NTM can cause various human diseases, with the most common disease being NTM pulmonary disease (PD) [3]. There are approximately 200 species of NTM, and the most common causative agent of PD is the *Mycobacterium avium* complex, with *Mycobacterium abscessus* (including subspecies *abscessus* or *massiliense*) being the second most common causative agent in some countries [1,4]. The incidence and prevalence of NTM-PD are increasing globally, leading to a significant increase in the disease burden [5].

Patients with NTM-PD can have heterogeneous and diverse clinical courses, with some cases naturally resolving without antibiotics, while others do not achieve treatment success despite aggressive antibiotic therapy [6,7]. In terms of detailed treatment guidelines, current global guidelines have recommended macrolide antibiotic-based multidrug treatment [8,9,10], and even after achieving consecutive culture-negative results, antibiotics should be maintained for 12 months to prevent recurrences [8,11,12,13,14]. Thus, a medical burden and associated adverse effects are inevitable. Moreover, despite these aggressive treatment strategies, treatment responses to antibiotics are not satisfactory, and approximately 30–40% of patients remain with treatment-refractory disease [15,16,17,18]. Therefore, there is a need for an objective, quantifiable biomarker that can predict disease progression or reflect severity in a comparable manner. However, to date, various clinical and experimental studies have made efforts to develop clinically useful biomarkers; unfortunately, no clinically useful biomarker has been developed thus far.

Serum carbohydrate antigen 19-9 (CA 19-9) is widely utilized as a tumor marker across various cancer types, with particular significance in pancreatic cancer research [19]. Interestingly, recent research findings have illuminated the potential elevation of CA19-9 in the context of chronic respiratory diseases, including bronchiectasis or pulmonary mycobacterial infections [20]. Furthermore, investigations have highlighted a notable decline in serum CA19-9 levels following antibiotic treatment in patients with NTM-PD, a phenomenon intricately linked to the severity of pulmonary computed tomography (CT) lesions [21,22,23]. Consequently, there is a growing interest in discerning the effective utilization of CA19-9 in the management of NTM-PD patients, which warrants further investigation. Despite these results, the clinical usefulness of CA 19-9 in NTM-PD remains unclear. To date, studies have not measured CA19-9 in a sufficient number of NTM-PD patients, or there have been no studies analyzing it based on treatment response criteria. Therefore, in this study, we compared the clinical characteristics of patients with and without elevated CA19-9 levels and investigated whether the increase in CA19-9 is related to the microbiological response to antibiotics in NTM-PD patients.

## 2. Methods

### 2.1. Study Population

**Figure 1:**
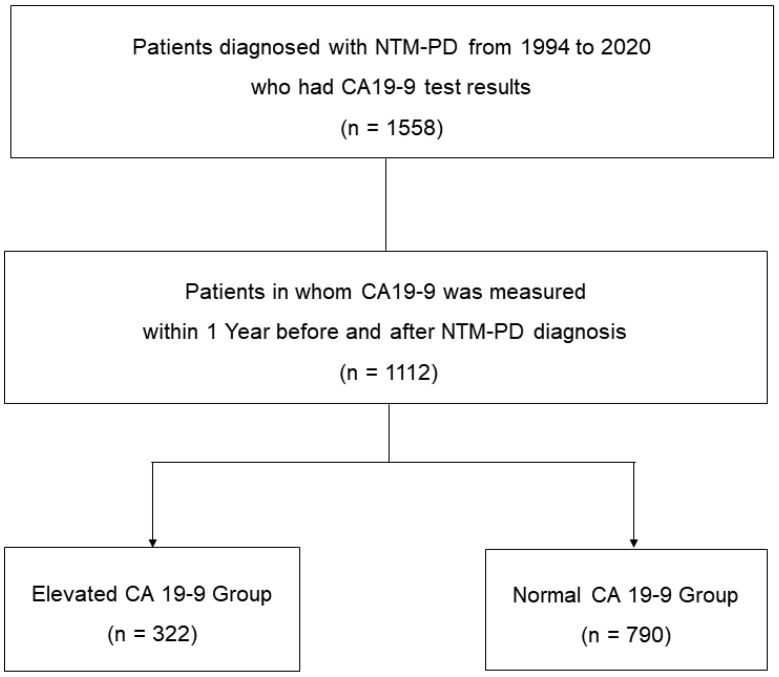
Study patients.

We retrospectively screened patients newly diagnosed with NTM-PD at Samsung Medical Center in Seoul, Republic of Korea, from January 1994 to December 2020. Most of the patients followed at our institution undergo routine health check-ups at intervals of 1 to 2 years, which include tumor marker blood tests such as CA19-9. During this period, we searched for patients diagnosed with NTM-PD who underwent CA19-9 blood testing, and we confirmed the presence of 1558 patients with NTM-PD who had received CA19-9 blood tests. Subsequently, we identified a total of 1112 individuals whose CA19-9 blood tests were conducted either within one year before or one year after the NTM-PD diagnosis (Figure 1). Therefore, we included these 1112 patients in the study analysis. The patients were categorized into two groups: the elevated CA19-9 (n = 322) group and the normal CA19-9 level (n = 790) group. We compared the clinical characteristics of these two groups, and additionally, we analyzed the association between microbiological outcomes after antibiotic treatment and CA19-9 levels among patients who initiated antibiotic therapy.

All patients met the NTM-PD diagnostic criteria. For January 1994 to December 2007, data were obtained from a retrospective cohort, and beginning in January 2008, data were obtained from an ongoing Institutional Review Board-approved prospective observational cohort (ClinicalTrials.gov Identifier: NCT00970801, IRB no. 2008-09-016) [24,25]. Informed consent was obtained from all participants.

### 2.2. Data Collection

We retrospectively collected baseline demographics, comorbidities, medical history, and laboratory and radiological findings of the study population at the time of NTM-PD diagnosis. Radiologically, the NTM-PD was categorized into nodular bronchiectatic (NB), fibrocavitary (FC), and non-classifiable forms according to chest computed tomography images. The NB form was defined by the presence of multifocal bronchiectasis and clusters of small nodules on chest computed tomography, regardless of the presence of small cavities in the lungs. The FC form was defined by the presence of cavitary opacities and pleural thickening. When the disease did not belong to either the FC or NB form, it was deemed non-classifiable [25].

NTM-PD severity was determined using the BACES score [body mass index < 18.5 kg/m^2^, age ≥ 65 years, presence of cavity, elevated erythrocyte sedimentation rate (ESR) in men > 15 mm/h and in women > 20 mm/h, and male sex; each worth one point]. One point was assigned for each item, and the total score was considered an indicator of mild (0–1 point), moderate (2–3 points), or severe (4–5 points) disease [26,27,28].

### 2.3. Measurement of CA 19-9 Levels

Serum CA 19-9 levels were measured through two distinct methods: firstly, using a COBAS e 801 analyzer (Roche Diagnostics, Mannheim, Germany) employing an electrochemiluminescence immunoassay, and secondly, utilizing a Dream Gamma-10 gamma counter (Shinjin Medics, Ilsan, Republic of Korea) which employed an immunoradiometric assay with a reference value of <37 U/mL. To ensure consistency in our study analysis, we established the reference value for CA 19-9 elevation as being ≥37 U/mL.

### 2.4. NTM Identification and Microbiological Response

Sputum was obtained for microbiological evaluation. Acid-fast bacilli smears and cultures were conducted using standard methods. NTM species were identified using polymerase chain reaction-restriction fragment length polymorphism analysis or reverse blot hybridization of the rpoB gene. Beginning in June 2014, species identification was carried out using a nested multiplex polymerase chain reaction and a reverse hybridization assay of the internal transcribed spacer region (AdvanSureTM Mycobacteria GenoBlot Assay; LG Life Sciences, Seoul, Republic of Korea).

‘Negative culture conversion’ was defined as at least three consecutive negative sputum cultures, collected at least 4 weeks apart based on the NTM-NET consensus statement [29]. The time to culture conversion was defined as the time to the date of the first negative culture. ‘Microbiological cure’ was defined as multiple consecutive negative cultures, but no positive cultures with the causative species after culture conversion until the end of anti-mycobacterial treatment.

### 2.5. Statistical Analysis

The statistical analyses were conducted using two software tools: SPSS software (IBM SPSS Statistics version 27, Chicago, IL, USA) and GraphPad Prism 9 (GraphPad, San Diego, CA, USA). The presentation of data was carried out comprehensively, utilizing either numbers and percentages or the interquartile range (IQR) to provide a thorough understanding of the dataset. For categorical variables, comparisons were made using either Pearson’s chi-square test or Fisher’s exact test, while continuous variables were subjected to comparison through the Mann–Whitney U test. In addition to these comparisons, correlation analysis was performed to explore the relationship between blood inflammatory tests and CA 19-9 levels. To visualize the cumulative culture conversion rates based on CA 19-9 levels, the Kaplan–Meier method was employed, and the results were compared using the log-rank test. Furthermore, to identify factors associated with microbiological cure within our study population, a multivariable logistic regression analysis was conducted. All tests were two-sided, with statistical significance set at *p* < 0.05.

## 3. Results

### 3.1. Comparison of the Characteristics of Patients with and without an Elevated CA19-9

The baseline clinical characteristics of the 1112 NTM-PD patients who had results of blood CA19-9 levels are shown in Table 1. The clinical characteristics of the study patients were collected at the time of NTM-PD diagnosis. Of the total, 61% were female, with a median age of 60 years. The majority (72%) were never-smokers. Bronchiectasis was observed in most patients (83%), and 37% had undergone past pulmonary tuberculosis treatment. In chest computed tomography scans, the majority (81%) of patients exhibited the NB form, while only 14% showed the FC form. Among all patients, 23% had cavitary lesions. *M. avium* complex (70%) was the most common causative organism.

Of all the study patients, 322 (29%) had elevated serum CA19-9 levels, while the remaining 790 (71%) had normal levels. When comparing the clinical characteristics of the two groups, there was a greater proportion of females and bronchiectasis in the group with elevated CA19-9 levels compared to the group with normal CA19-9 levels. Notably, the levels of blood ESR, C-reactive protein (CRP), and the rate of initiation of antibiotic treatment differed significantly between the two patient groups. ESR and CRP were higher in the elevated CA19-9 group (*p* < 0.001 and *p* = 0.029, respectively), as was the antibiotic initiation rate (74% vs. 57%, *p* < 0.001). For the 811 patients for whom ESR data were available, serum CA19-9 levels were weakly positively correlated with ESR (Spearman’s rank correlation coefficient, ρ = 0.139, *p* < 0.001). However, there was no significant difference in the proportion of BACES severity between the group with elevated CA19-9 levels and the group of patients with normal levels (*p* = 0.842).

### 3.2. Microbiological Response in Patients Who Received Antibiotic Therapy

We evaluated the microbiological response based on CA19-9 levels in 688 NTM-PD patients who received antibiotic treatment. Among these 688 patients, 239 (35%) had elevated serum CA19-9 levels, while the remaining 449 (65%) had normal levels. The microbiological response after antibiotic therapy in patients who received antibiotics is shown in Table 2. The median duration of antibiotic therapy for the elevated CA19-9 group and the normal CA19-9 group was 19.0 (IQR 15.2–24.5) months and 19.6 (15.2–24.6) months, respectively, with no statistical difference (*p* = 0.949).

**Figure 2:**
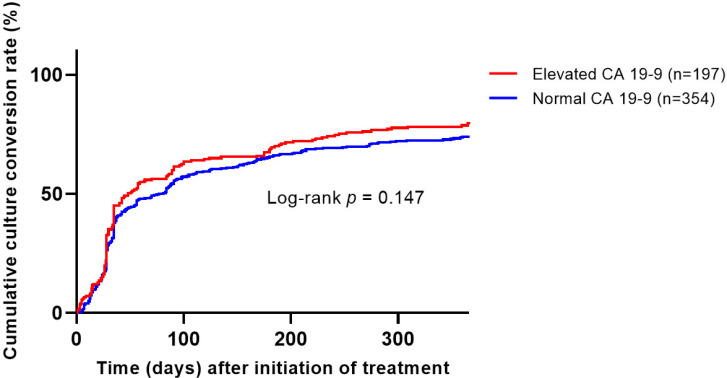
Cumulative culture conversion rate according to CA19-9 elevation (Kaplan–Meier log-rank test, n = 551, *p* = 0.147).

The rate of culture conversion within one year after starting antibiotics showed no statistical difference between the CA19-9 elevation group and the normal group (80% versus 72%, *p* = 0.055). Of the 551 patients who completed antibiotics and were eligible for microbiological cure evaluation, 466 (85%) achieved microbiological cure. Among them, 89% (175 of 197) of the CA19-9 elevation group and 82% (291 of 354) of the normal group achieved microbiological cure, with a slightly higher tendency in the CA19-9 group (*p* = 0.039). However, when assessing the cumulative culture conversion rate in patients eligible for microbiological cure evaluation, no significant difference was observed between the two groups based on CA19-9 elevation status (Kaplan–Meier, log-rank test, *p* = 0.147, Figure 2). Additionally, the serum CA19-9 levels did not differ between those who achieved microbiological cure and those who did not (Supplementary Figure S1).

### 3.3. Factors Associated with Microbiological Cure including CA 19-9 Levels

To assess the impact of serum CA19-9 on microbiological cure, multivariate analysis was conducted. The analysis was performed on the subset of 434 individuals for whom adjusted variable analysis was possible, including other factors along with CA19-9 level (Table 3). Current smoking, bronchiectasis, acid-fast bacilli smear positivity, and the *M. abscessus* strain demonstrated a statistically significant negative impact on microbiological cure. In contrast, the *M. massiliense* strain was found to have a significantly positive association with microbiological cure (adjusted odds ratio 3.108, *p* = 0.040). However, the serum CA19-9 level did not show a statistically significant association with microbiological cure in both univariate (odds ratio 1.002, 95% confidence interval 0.999–1.005, *p* = 0.262) and multivariate analyses (odds ratio 1.004, 95% confidence interval 0.999–1.008, *p*= 0.121).

## 4. Discussion

This study compared the clinical characteristics between NTM-PD patients with elevated serum CA19-9 levels and those with normal CA19-9 levels, and investigated whether the elevated CA19-9 level can influence the microbiological response to antibiotic therapy in NTM-PD patients. Although the serum CA19-9 level had positive associations with inflammatory markers such as blood levels of ESR and CRP, the CA19-9 level was not associated with the microbiological response in multivariable analysis after adjusting for various clinical factors. Thus, our data suggest that, while serum CA19-9 may reflect the extent of inflammation in NTM-PD patients with elevated CA19-9 levels, as evident from inflammatory markers, it has a limited role as a predictor of antibiotic treatment outcomes in NTM-PD patients requiring treatment.

When we initially planned this study, our expectation was that higher serum CA19-9 levels would be linked to poorer treatment responses in NTM-PD patients, based on previous research indicating a positive correlation between serum CA19-9 levels and the extent of lung lesions observed in chest CT scans [22]. However, contrary to our initial expectations, our analysis revealed that elevated serum CA19-9 levels did not result in worse microbiological responses to antibiotic treatment. Moreover, there were no discernible clinical characteristics that clearly distinguished individuals with elevated CA19-9 levels from those with normal levels, except for inflammatory markers such as ESR or CRP. These findings suggest that CA19-9 is unlikely to serve as a specific biomarker associated with the pathogenesis of NTM-PD. Thus, our data suggest that the presence of elevated serum CA19-9 levels at the time of NTM-PD diagnosis does not necessarily indicate a poor treatment outcome or the need for more aggressive antibiotic therapy. Thus, we found that CA19-9 is not useful as a biomarker. Especially in a situation where the clinical significance of CA19-9 was not clearly established, there were lingering questions about whether to administer antibiotics more aggressively or for an extended duration when CA19-9 levels were elevated in NTM-PD patients. Additionally, there may be uncertainties about whether antibiotic treatment should continue until CA19-9 returns to normal values through antibiotic therapy. In these contexts, we believe that our research data has clinical significance because it demonstrates that the elevation of CA19-9 in NTM-PD patients does not indicate a poor treatment response.

In terms of the clinical utility of CA19-9 in NTM-PD, studies have shown a decrease in CA19-9 levels following antibiotic treatment for NTM-PD [21,23]. When interpreting the research results analyzed by our research team in conjunction with the aforementioned previous studies, elevated CA19-9 levels in NTM-PD patients suggest that it may be a more useful indicator for monitoring treatment responses or disease progression, rather than for predicting treatment outcomes. In real-world clinical practice, monitoring the treatment response in NTM-PD is particularly challenging. There is no easily discernible indicator during antibiotic treatment to determine whether a patient’s bacterial load is decreasing or if the disease is improving. Some tests, such as the acid-fast bacilli culture or quantitative culture during antibiotic therapy, can take several weeks to confirm results. Additionally, clinical symptoms of patients can sometimes be ambiguous, and respiratory symptoms can occur due to underlying lung conditions like bronchiectasis rather than NTM-PD. Moreover, detecting subtle changes in bronchiolitis lesions or small cavity formation through simple chest radiography is difficult. In these aspects, CA19-9 can serve as a valuable tool. Furthermore, given that NTM-PD can have a recurrence rate of up to 30% [30], if an increase in CA19-9 after treatment completion can predict relapse, it can also play a role as a biomarker for predicting relapse. However, no research has examined whether an increase in CA19-9 levels after completing treatment for NTM-PD is associated with relapse. Thus, we believe that further studies are required to determine whether CA19-9 can be used as a biomarker for monitoring the treatment response or predicting relapse.

In our study, there was no significant difference in the distribution of BACES severity between the elevated CA19-9 group and the normal CA19-9 group. This finding also indicates that CA19-9 does not well reflect the long-term prognosis of patients. BACES severity was originally developed as an indicator that reflects the survival rates of NTM-PD patients [26]. It is known that BACES severe patients have a higher mortality rate compared to BACES mild patients, and this finding has been consistent not only in South Korea study but also in recent validation studies conducted in Canada [27,28,31]. Furthermore, recent studies have reported that treatment responses vary depending on the severity of BACES, with BACES severe patients showing lower cure rates compared to those with BACES mild [27,28]. Therefore, in our study, the lack of association between patient distribution based on BACES severity and the elevation of CA19-9 suggests once again that a rise in CA19-9 has limited relevance to the long-term prognosis of NTM-PD patients.

Our study has several limitations. First, our participants were primarily patients who had CA19-9 measurements as part of routine health check-ups, which could introduce selection bias as CA19-9 was not measured consecutively in a prospective manner. In South Korea, regular health check-ups are conducted at intervals of several years through the National Health Insurance, and during these check-ups, tumor marker tests such as CA19-9 are often performed. However, there may be NTM-PD patients being monitored at our institution who have not undergone health check-ups or have not had CA19-9 tests. Therefore, we believe that further prospective research is needed to re-evaluate this matter in the future. Second, we established the evaluation time for CA19-9 to be within one year from the time of NTM-PD diagnosis, which could also introduce bias. However, since CA19-9 is not a mandatory blood test for all NTM-PD patients, we conducted the study with this criterion chosen arbitrarily. Third, we did not evaluate the clinical significance of CA19-9 in patients who had not undergone antibiotic treatment. Approximately 15–30% of NTM-PD patients experience spontaneous culture conversion and recover naturally without antibiotics [6]. However, we did not assess whether the rate of such spontaneous culture conversion is higher in patients with normal CA19-9 levels. The reason for this is that stable patients who do not undergo antibiotic treatment often have mild symptoms, making it challenging to conduct regular and prolonged follow-up tests. Therefore, we believe that further research is necessary to address this issue. Finally, it should be noted that some patients did not undergo comprehensive testing to identify potential underlying conditions that might lead to an elevation in CA19-9 levels, such as gastrointestinal malignancies.

In conclusion, our data have revealed a positive association between serum CA19-9 levels and the inflammatory markers ESR and CRP in NTM-PD patients. However, the CA19-9 level was not associated with the microbiological response to antibiotic treatment. Therefore, CA19-9 has a limited role as a predictor of antibiotic treatment outcomes.

## Figures and Tables

**Table 1:** Baseline characteristics of study patients.

Characteristics	Total (n = 1112)	Elevated CA 19-9 (n = 322)	Normal CA 19-9 (n = 790)	*p*-Value
Sex, female	677 (61)	228 (71)	449 (57)	<0.001
Age, year	60 (52–68)	60 (53–70)	60 (52–67)	0.190
Body mass index, kg/m^2^	20.7 (19.2–22.5)	20.3 (18.8–21.9)	20.9 (19.3–22.9)	0.001
Smoking status				0.001
Never	799 (72)	256 (80)	543 (69)	
Ex	283 (25)	62 (19)	221 (28)	
Current	30 (3)	4 (1)	26 (3)	
Comorbidity				
Bronchiectasis	922 (83)	299 (93)	623 (79)	<0.001
Previous pulmonary tuberculosis	414 (37)	121 (38)	293 (37)	0.878
Other malignancy	172 (16)	39 (12)	133 (17)	0.048
Obstructive lung disease	82 (7)	18 (6)	64 (8)	0.146
Chronic pulmonary aspergillosis	24 (2)	5 (2)	19 (2)	0.375
Idiopathic pulmonary fibrosis	12 (1)	5 (2)	7 (1)	0.344
Lung cancer	12 (1)	3 (1)	9 (1)	1.000
Positive sputum AFB smear	482 (43)	143 (44)	339 (43)	0.647
Radiological form				<0.001
Nodular bronchiectatic form	902 (81)	297 (92)	605 (77)	
Fibrocavitary form	154 (14)	20 (6)	134 (17)	
Non-classifiable form	56 (5)	5 (2)	51 (7)	
Cavity	259 (23)	64 (20)	195 (25)	0.085
Etiology				
*M. avium* complex	797 (72)	209 (65)	588 (74)	0.001
*M. massiliense*	109 (10)	37 (12)	72 (9)	0.227
*M. abscessus*	103 (9)	45 (14)	58 (7)	0.001
Mixed	59 (5)	21 (7)	38 (5)	0.248
Others	44 (4)	10 (3)	34 (4)	0.353
Laboratory test				
ESR, mm/h (n = 811)	27 (15–47)	31 (18–54)	26 (14–45)	<0.001
CRP, mg/dL (n = 906)	0.13 (0.05–0.68)	0.16 (0.06–0.68)	0.12 (0.05–0.70)	0.029
Initiation of antibiotic treatment	688 (62)	239 (74)	449 (57)	<0.001
BACES severity score (n = 811) ^¶^				0.842 *
Mild	360 (44)	116 (46)	244 (44)	
Moderate	372 (46)	115 (45)	257 (46)	
Severe	79 (10)	23 (9)	56 (10)	

Data are presented as number (%) or median (interquartile range). * *p* for trend = 0.561. AFB = acid-fast bacilli, ESR = erythrocyte sedimentation rate, CRP = C-reactive protein, BACES = Body mass index, Age, Cavity, ESR, and Sex (each one point). ^¶^ Patients were classified into the three groups according to their scored severity: mild (0–1 point), moderate (2–3 points), and severe (4–5 points).

**Table 2:** Microbiological response in patients who received antibiotic treatment (n = 688).

Variables	Total (n = 688)	Elevated CA 19-9 (n = 239)	Normal CA 19-9 (n = 449)	*p*-Value
Treatment duration, month	19.4 (15.2–24.5)	19.0 (15.2–24.5)	19.6 (15.2–24.6)	0.949
Culture conversion, within one year	437/583 (75)	164/206 (80)	273/377 (72)	0.055
Microbiological cure	466/551 (85)	175/197 (89)	291/354 (82)	0.039
Time to culture conversion, month ^¶^	1.2 (0.9–5.0)	1.4 (0.9–5.8)	1.2 (0.9–4.5)	0.338

Data are presented as number (%) or median (interquartile range). ^¶^ Symbol refers to the assessment of those who achieved microbiological cure.

**Table 3:** Factors associated with microbiological cure in study patients who received antibiotic treatment (n = 434).

Variables	Univariate Analysis		Multivariate Analysis	
	Odd Ratio (95% Confidence Interval)	*p*-Value	Adjusted Odd Ratio (95% Confidence Interval)	*p*-Value
Sex, female	1.934 (1.209–3.095)	0.006	–	–
Age, year	0.985 (0.964–1.006)	0.162	–	–
Body mass index, kg/m^2^	1.043 (0.948–1.147)	0.389	–	–
Smoking status				
Never	reference	–	reference	–
Ex	0.625 (0.369–1.059)	0.081	0.617 (0.313–1.216)	0.163
Current	0.395 (0.120–1.301)	0.127	0.159 (0.037–0.686)	0.014
Comorbidities				
Bronchiectasis	0.748 (0.338–1.439)	0.384	0.358 (0.153–0.837)	0.018
Obstructive lung disease	1.250 (0.545–2.867)	0.598	–	–
Chronic pulmonary aspergillosis	1.285 (0.287–5.761)	0.743	–	–
Idiopathic pulmonary fibrosis	1.281 (0.156–10.547)	0.818	–	–
Previous pulmonary tuberculosis	0.603 (0.378–0.960)	0.033	–	–
Positive sputum AFB smear	0.370 (0.223–0.615)	<0.001	0.289 (0.155–0.541)	<0.001
Cavity	0.836 (0.514–1.360)	0.471	–	–
Etiology				
*M. avium* complex	1.364 (0.846–2.199)	0.203	–	–
*M. massiliense*	3.520 (1.249–9.917)	0.017	3.108 (1.055–9.150)	0.040
*M. abscessus*	0.329 (0.172–0.629)	0.001	0.329 (0.147–0.735)	0.007
Laboratory findings				
ESR, mm/h	1.004 (0.995–1.014)	0.367	–	–
CRP, mg/dL	0.918 (0.809–1.043)	0.189	–	–
CA 19-9 (U/mL)	1.002 (0.999–1.005)	0.262	1.004 (0.999–1.008)	0.121
BACES severity score ^¶^				
Mild	reference	–	reference	–
Moderate	0.838 (0.492–1.426)	0.514	–	–
Severe	0.563 (0.233–1.358)	0.201	–	–

Data are presented as median (interquartile range). AFB = acid-fast bacilli, ESR = erythrocyte sedimentation rate, CRP = C-reactive protein, BACES = Body mass index, Age, Cavity, ESR, and Sex (each one point). ^¶^ Patients were classified into the three groups according to their scored severity: mild (0–1 point), moderate (2–3 points), and severe (4–5 points).

## Data Availability

All relevant data are within the paper.

## Supplementary Materials

The following supporting information can be downloaded at: https://www.mdpi.com/article/10.3390/jcm12247751/s1, Figure S1: Comparison of CA 19-9 level between microbiological cure (n = 466) and failure (n = 85) groups (total n = 551)
