# Supplementary material for: The Clinical Implications of Serum Carbohydrate Antigen 19-9 Levels in Patients with Nontuberculous Mycobacteria Pulmonary Disease
Source: J Clin Med. 2023 Dec 18;12(24):7751. doi: 10.3390/jcm12247751 (PMC10743919; doi:10.3390/jcm12247751)

[Supplementary Data]

The Clinical Implications of Serum Carbohydrate Antigen 19-9 Levels in Patients with Nontuberculous Mycobacterial Pulmonary Disease

Daegeun Lee[1], Byung Woo Jhun[2]

[1]Division of Pulmonary, Allergy and Critical Care Medicine, Chung-Ang University Gwangmyeong Hospital, Gwangmyeong, South Korea

[2]Division of Pulmonary and Critical Care Medicine, Department of Medicine, Samsung Medical center, Sungkyunkwan University School of Medicine, Seoul, Republic of Korea

Supplementary Figure S1. Analysis of the correlation between serum CA19-9 levels and ESR levels (n = 811).

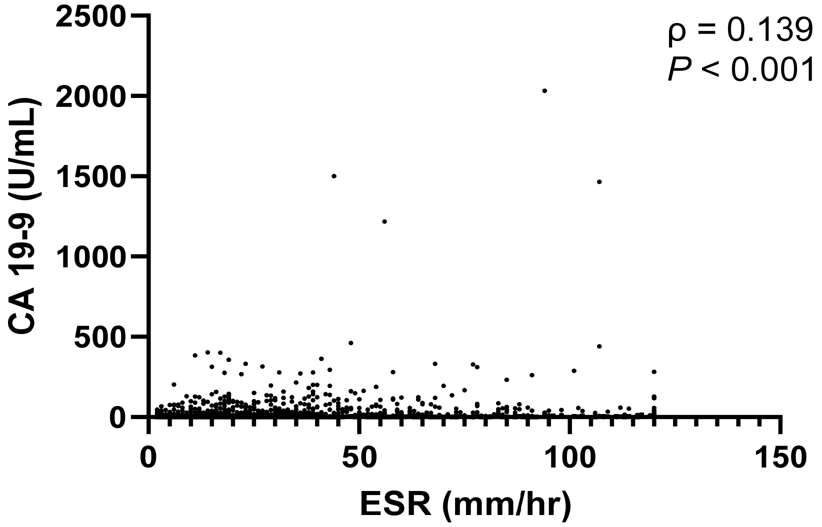

Supplementary Figure S2. Comparison of CA 19-9 level between microbiological cure (n = 466) and failure (n = 85) groups (total n = 551)

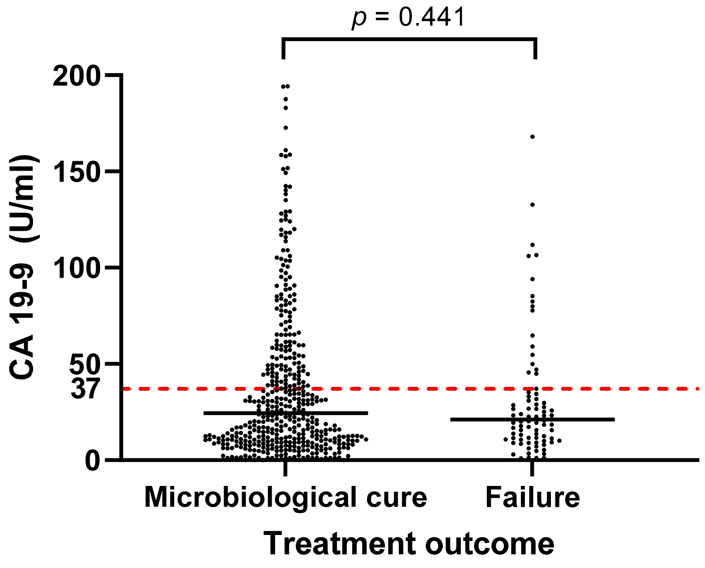

Supplement: Supplementary file 1 [file jcm-12-07751-s001.zip › jcm-2711532-supplementary.pdf]
